# *NF2*: An underestimated player in cancer metabolic reprogramming and tumor immunity

**DOI:** 10.1038/s41698-024-00627-5

**Published:** 2024-06-15

**Authors:** Duo Xu, Shiyuan Yin, Yongqian Shu

**Affiliations:** Department of Oncology, The First Affiliated Hospital of Nanjing Medical University, Nanjing, China

**Keywords:** Molecular medicine, Health sciences

## Abstract

Neurofibromatosis type 2 (*NF2*) is a tumor suppressor gene implicated in various tumors, including mesothelioma, schwannomas, and meningioma. As a member of the ezrin, radixin, and moesin (ERM) family of proteins, merlin, which is encoded by *NF2*, regulates diverse cellular events and signalling pathways, such as the Hippo, mTOR, RAS, and cGAS-STING pathways. However, the biological role of *NF2* in tumorigenesis has not been fully elucidated. Furthermore, cross-cancer mutations may exert distinct biological effects on tumorigenesis and treatment response. In addition to the functional inactivation of *NF2*, the codeficiency of other genes, such as cyclin-dependent kinase inhibitor 2A/B (*CDKN2A/B*), BRCA1-associated protein-1 (*BAP1*), and large tumor suppressor 2 (*LATS2*), results in unique tumor characteristics that should be considered in clinical treatment decisions. Notably, several recent studies have explored the metabolic and immunological features associated with *NF2*, offering potential insights into tumor biology and the development of innovative therapeutic strategies. In this review, we consolidate the current knowledge on *NF2* and examine the potential connection between cancer metabolism and tumor immunity in merlin-deficient malignancies. This review may provide a deeper understanding of the biological roles of *NF2* and guide possible therapeutic avenues.

## Introduction

The neurofibromatosis type 2 (*NF2*) gene is a tumor suppressor located at chromosomal region 22q12 and is involved in the development of various human malignancies, including mesothelioma, schwannomas, and meningioma^1^. Tumor suppressor genes (TSGs) cannot be directly targeted due to their functional inactivation, which poses a challenge for clinical treatment. Emerging evidence has shown that indirectly targeting downstream activation signals driven by TSG inactivation can specifically kill tumor cells through a process known as “synthetic lethality”. The abovementioned strategy has been widely used for treating *NF2*-driven tumors, but few efficient treatments have been developed. Notably, cross-cancer mutation patterns have gradually attracted increased amounts of attention due to their influence on tumor behavior and treatment response, highlighting that focusing solely on one mutation has not proven enough to help identify promising treatments. Therefore, in this review, we summarize the cross-cancer mutation patterns and subsequent biological alterations in *NF2*-related tumors. Nevertheless, previous studies have reported that *NF2* plays pivotal roles in cell contact inhibition, mitogenic signal transduction inhibition, proteolysis, epithelial adhesion, and polarity^2–7^, but the biological roles of *NF2* in tumor metabolism and immunity have not been systematically discussed. We also explored the current understanding of how *NF2* deficiency drives tumorigenesis from the perspective of cancer metabolism reprogramming and antitumour immunity. Finally, we discuss the potential underlying metabolic and immunological networks involved in *NF2*-driven malignancies, aiming to highlight new perspectives on the treatment of *NF2*-deficient tumors.

## Genetic alterations in *NF2*-related tumors

### Mutations of *NF2* gene in patients

**Fig. 1:**
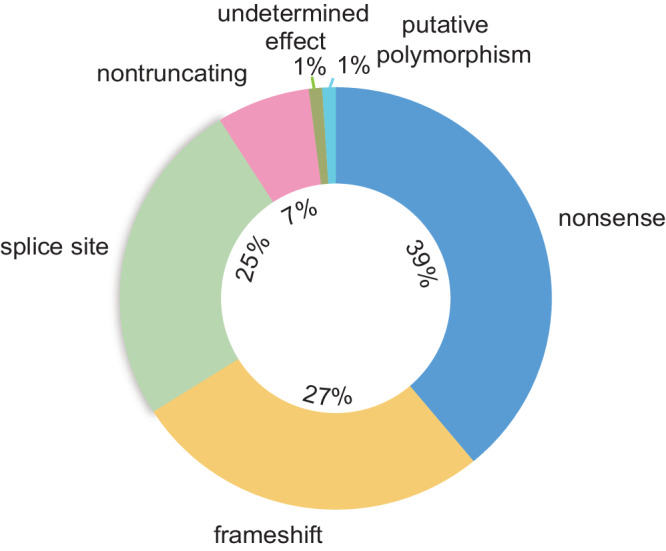
Genetic mutation spectrum of *NF2.* In research correlated with neurofibromatosis type 2 patients, genetic alterations in the *NF2* gene could be separated into three subsets: small alterations, multiexonic alterations, whole-gene deletions, and chromosomal rearrangements. Small alterations were found to be most common among these three subtypes. The data presented in this chart were obtained from Ahronowitz, I., et al., Mutational spectrum of the *NF2* gene: a meta-analysis of 12 years of research and diagnostic laboratory findings. *Hum Mutat* **28**, 1–12 (2007).

Emerging evidence has shown that inactivation of *NF2* mainly results from recurrent gene fusions and splice alterations. These fusions tend to be repulsive to other genomic alterations, and alternative splicing contributes to the generation of multiple *NF2* transcripts^8,9^. In alignment with these findings, variable *NF2* transcripts were observed in patients with neurofibromatosis type 2, as well as in patients with mesothelioma^10,11^. An analysis of constitutional alterations in *NF2*-mutant patients revealed that nonsense (39%) and frameshift (27%) mutations constitute the majority of slight alterations and are closely associated with a more severe disease phenotype characterized by an increased frequency of multiple and recurring meningiomas as regard to patients with neurofibromatosis type 2^12,13^. In contrast, splice site (25%) and nontruncation (7%) mutations make up a smaller fraction of the total population (Fig. 1)^13^. In addition to wild-type patients, mesothelioma patients also exhibit multiple *NF2* transcripts, including truncated transcripts, splicing variants and unexpected variants^11^. Notably, targeted therapy for specific *NF2* mutations is unavailable, which is partly due to the lack of hotspot mutations.

### *NF2* mutations and human cancer

#### Malignant mesothelioma

The link between *NF2* and mesothelioma was first identified in 1995 when Sekido et al. detected 7 mutations in 17 mesothelioma patients, suggesting the participation of *NF2* in mesothelioma tumorigenesis^14^. The current exploration of *NF2* in the field of mesothelioma has focused mostly on pleural mesothelioma, which represents approximately 85% of all malignant mesothelioma (MM) cases^15^. Malignant pleural mesothelioma (MPM) is an aggressive tumor with a poor prognosis and is closely associated with asbestos exposure^16^. Approximately 30–50% of somatic mutations in *NF2* occur in pleural mesotheliomas^17–19^. In hemizygous *Nf2*^KO3/+^ FVB/N mice, inactivation of *Nf2* contributes to the formation of mesothelioma after asbestos exposure^20^. Moreover, even without asbestos exposure, *NF2* mutation together with the loss of other TSGs could induce the development of MM, indicating that loss of the *NF2* locus is critical^21^. Paradoxically, after conducting exome sequencing of multiregional tumor samples, evidence has shown that loss of *NF2*/22q is preferred as a late clonal event in mesothelioma^22,23^. Therefore, identifying the exact function of *NF2* in tumorigenesis is worthwhile. Histologically, MPM can be divided into three subtypes: epithelioid, sarcomatoid and biphasic^24^. Intriguingly, genetic alterations in *NF2* have frequently been observed in biphasic and sarcomatoid types, indicating that *NF2* inactivation might be involved in epithelial–mesenchymal transition^25–27^. The current chemotherapeutic approach for MPM, regardless of the presence of an *NF2* mutation, is based on a combination of platinum and pemetrexed, with bevacizumab added because of its high refractoriness to conventional therapies^28^. Therapies targeting focal adhesion kinase (FAK), the Hippo pathway, mechanistic target of rapamycin (mTOR), statins and cyclooxygenase 2 (COX-2), as well as immunotherapy, are currently being explored for the treatment of merlin-negative mesothelioma^18^. Regarding clinical diagnosis, immunohistochemistry (IHC) of *NF2* with D1D8 and D3S3 W antibodies has been documented as a promising method for distinguishing between benign and malignant mesothelial processes. However, replications on a large number of samples are needed to validate this interesting point^29^.

#### Meningioma

In meningioma, *NF2* is the most common point mutation gene and is significantly different in mutation probability (49%) compared to other molecules (<10%)^30^. Moreover, the mutation rate of *NF2* was positively correlated with meningioma grade. Unlike sporadic mutations, which include smoothened (SMO), tumor receptor-associated factor 7 (TRAF7) and phosphatidylinositol-4,5-bisphosphate 3 kinase catalytic subunit α (PIK3CA), *NF2* mutations are more likely to occur in younger individuals and produce multiple meningiomas^31^. Due to the lack of effective treatments, surgical excision and radiosurgery currently dominate the treatment of meningiomas^30,31^. Notably, Nassiri et al. classified meningiomas into four main types based on molecular subtype: immunogenic (MG1), benign (wild-type) NF2 (MG2), hypermetabolic (MG3), and proliferative (MG4). Among these, MG1 meningioma harbours a uniform loss of chromosome 22q and concurrent *NF2* point mutations, resulting in biallelic *NF2* inactivation. Intriguingly, MG1 tumors are closely associated with tumor immunity, as evidenced by increased macrophage infiltration, as well as the involvement of B cells, platelets, and cytokines such as IL-6 and interferon-gamma (IFN-γ), as illustrated by the detection of protein abundance^30^. Taken together, these findings may lead to the development of immunotherapy for *NF2*-associated meningiomas.

#### Vestibular schwannomas

Genetic alterations in *NF2* are likely the basis for neurofibromatosis type 2, which is characterized by the formation of vestibular schwannomas. Approximately 70-90% of patients with *NF2* mutations develop bilateral vestibular schwannomas (VSs)^32,33^. In sporadic vestibular schwannomas, cells with dysfunction of both *NF2* alleles, which is caused mainly by mutation and allelic loss, exhibit an increased proliferation rate compared with tumors with a single mutation^34^, further indicating the indispensable role of *NF2* in the occurrence of neurofibromatosis. Currently, gamma knife radiosurgery is a well-accepted treatment for VS. For *NF2*-associated VS resistant to radiotherapy, targeted therapy such as bevacizumab has been shown to cause 30-60% tumor shrinkage and 20% hearing improvement^35,36^, although it has several side effects, such as apparent drug resistance and rebound tumor progression^37^. Other therapies targeting erythroblastic oncogene B (ErbB) receptors (such as trastuzumab, lapatinib, and erlotinib), platelet-derived growth factor receptor (PDGFR), or the phosphoinositide 3-kinase (PI3K)-AKT pathway have shown decreased efficacy or controversial results in patients with *NF2*-related VS^38^.

#### Other tumors

**Fig. 2:**
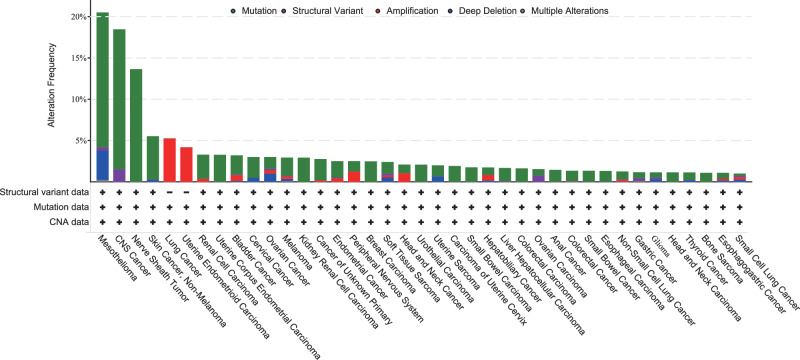
The mutation rate of *NF2* in the pancancer cohort. The top three cancers with the highest mutation rates of *NF2* are mesothelioma, CNS cancer, and nerve sheath tumors. The data presented in this chart were obtained from the China Pancancer (OrigiMed, Nature 2022), MSK-IMPACT Clinical Sequencing Cohort (MSK, Nat Med 2017), Cancer Therapy and Clonal Hematopoiesis (MSK, Nat Genet 2020), MSK MetTropism (MSK, Cell 2021), and Pancancer Analysis of Whole Genomes (ICGC/TCGA, Nature 2020) cohorts, and downloaded from the cBioPortal.

As a tumor suppressor gene associated with cancer development, *NF2* mutations are also observed in various other human malignancies, including melanoma, clear cell renal cell carcinoma, breast cancer, hepatobiliary cancer, glioblastoma, medullary thyroid carcinoma, and prostate cancer. Compared to those of mesothelioma and neurologic tumors mentioned earlier, the incidences of *NF2* mutations in common human cancers are markedly lower: 4.5% in breast cancer, 4.5–8.3% in colorectal cancer, 5% in melanoma, approximately 2.2% in hepatocellular cancer, 2.2% in acute myelogenous leukemia, and 2.2% in squamous cell lung carcinomas (Fig. 2)^39–41^. In hepatobiliary cancer, merlin was shown to be associated with liver progenitor cells and tumor development, and in prostate cancer, a highly invasive and chemoresistant state related to merlin deficiency was observed. Furthermore, the risk of recurrence was also shown to be elevated in *NF2*-related medullary thyroid carcinoma^1^. These findings led to the consideration of the function of merlin and its underlying mechanisms in other human malignancies, which deserves further exploration.

## Cellular roles of *NF2* in cancer

### Merlin––*NF2* gene production

Merlin (moesin-ezrin-radixin-like protein) is a protein encoded by the *NF2* gene. It is a member of the ezrin, radixin, and moesin (ERM family of proteins) families and links F-actin, transmembrane receptors, and intracellular signalling molecules. It consists of three main structural domains: an amino-terminal protein 4·1-ezrin-radixin-moesin (FERM) domain in the N-terminus, an alpha-helical domain in the middle, and a carboxyterminal domain (CTD) in the C-terminus^42^. The molecular conformation of merlin undergoes a change when the S518 residue is phosphorylated by protein kinase A (PKA) or p21-activated kinase (PAK) or when it is dephosphorylated by the myosin phosphatase-1 protein phosphatase-1δ (MYPT1-PP1δ). This alteration leads to interactions between the head and tail domains of merlin, allowing it to transition between an open and a closed conformation^43^, a potential basis for functioning as a tumor suppressor^17^. The close conformation of dephosphorylated merlin is involved in tumor suppression via its active form. Merlin is expressed not only in the plasma membrane and cytoskeleton but also in the nucleus and engages in several signalling pathways, such as the Hippo–YAP, PI3K-Akt-mTOR, and RAS pathways^1,6^.

### Signalling pathways related to *NF2*/merlin

#### Hippo-YAP

**Fig. 3:**
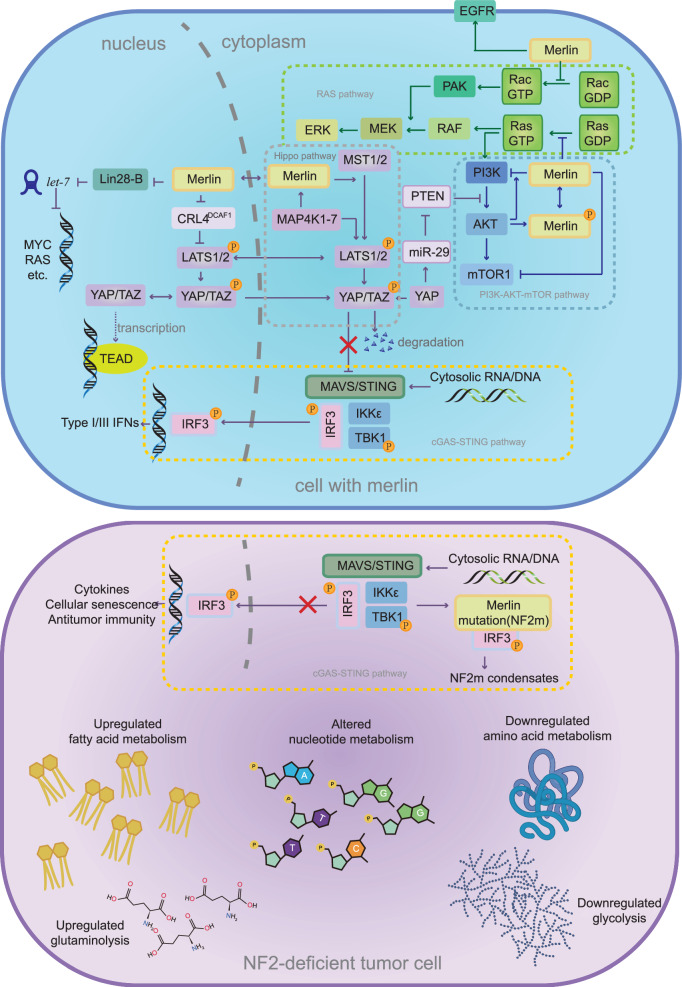
The interplay between *NF2*-related downstream signalling pathways and tumor metabolism. Several canonical downstream signalling pathways of *NF2*, including the Hippo, PI3K-AKT-mTOR, RAS, and cGAS-STING pathways, participate in cancer metabolic reprogramming. Created with BioRender.com.

The Hippo pathway is implicated in many aspects of tumors, including organ development, tissue regeneration, and epithelial-to-mesenchymal transition (EMT)^44^. Merlin negatively regulates the Hippo pathway in the cytoplasm and the nucleus. On the one hand, STE20-like protein kinases (MST1/2, Hippo kinases) phosphorylate LATS1/2 on the plasma membrane, which is mediated by merlin^45^. In turn, LATS1/2 phosphorylate the downstream effector Yes-associated protein (YAP) and its paralogue, WW domain-containing transcription regulator 1 (TAZ), blocking their role as transcriptional coactivators of transcription factors (TFs), including those in the TEAD (Transcriptional Enhanced Associate Domain) family^46^. Conversely, by binding to the E3 ubiquitin ligase CRL4^DCAF1^, merlin inhibits the function of this gene in the nucleus. CRL4^DCAF1^ can promote LATS1 polyubiquitylation and LATS2 oligoubiquitylation, which leads to the inactivation of LAST1 and LAST2 and thus the activation of YAP-driven transcription (Fig. 3)^6^. Additionally, the understanding of the Hippo pathway is evolving. Through weighted gene coexpression network analysis (WGCNA), Yang et al. reported that genetic alteration of *NF2* in MPM patients has a subtle impact on the expression of phospho-YAP (S127), suggesting that merlin may play an additional role independent of the classical Hippo–YAP pathway^47^. Moreover, an updated model revealed two generally independent signalling modules, MST1/2-SAV1-WWC1-3 (HPO1) and MAP4K1-7-NF2 (HPO2), where MAP4K1-7, a Hippo-like kinase, can phosphorylate and activate LATS1/2 and merlin^48^. These two signalling modules coregulate the activity of LATS1/2 kinase and YAP/TAZ but were found to differentially regulate liver size and liver cancer development in mice^49^. The specific mechanisms through which *NF2* is targeted through the Hippo pathway have not been fully explored, and further studies are needed.

The targeting of YAP activity by verteporfin (an inhibitor of the YAP-TEAD interaction) has been validated in MPM cells^50^. However, further exploration and validation are needed for clinical applications. Regarding inflammation, merlin influences YAP in the Hippo pathway, which promotes the transcription of COX-2. COX-induced PGI2 may play a role in the tumor microenvironment (TME) of *NF2*-deficient VSs. However, no significant effect of aspirin was observed in a study of VS patients^51^. Similarly, celecoxib also failed to inhibit *NF2*-associated VSs^38,52^. Upon cell‒cell contact, merlin physically restricts epidermal growth factor receptor (EGFR) internalization, further disrupting EGFR signalling. Moreover, sustained EGFR activation was observed in *NF2*-deficient cells^2^. However, several EGFR-TKIs have little effect on *NF2*-related tumors. Interestingly, evidence has shown that YAP is a crucial mediator of EMT-mediated resistance to EGFR-targeted therapies^47,53,54^.

#### PI3K-AKT-mTOR pathways

In neurofibromatosis and meningioma, evidence shows that loss of merlin promotes activation of the PI3K-AKT-mTOR pathway, leading to Schwann cell proliferation^55^. At the level of PI3K, merlin can inhibit its activity by preventing the long form of the PI3K enhancer (PIKE-L) from binding to PI3K, which in turn affects downstream signalling^56,57^. In addition, the interaction between merlin and PIP3 was significantly enhanced by AKT. However, AKT phosphorylates merlin inversely, and this phosphorylation inhibits the proapoptotic effect of merlin as well as its subcellular distribution and cell migration (Fig. 3)^58^. Inactivation of merlin in cells that have lost their anchorage to the extracellular matrix rescues mTORC1 signalling, suggesting that depletion of merlin contributes to the upregulation of mTORC1 expression^59^. Similar effects of merlin on mTOR1 were noted in neurological tumors (e.g., meningioma cells and arachnoid cells) both in vitro and in vivo^60^ and were found to be independent of the PI3K-AKT and MAPK/ERK pathways^59,60^. It is possible that this AKT-independent mechanism affects cell-to-cell contact inhibition^61^. Merlin can act on CRL4, a ubiquitin ligase that can degrade TSC2 (an inhibitor of mTOR). Merlin may inhibit mTOR activity through CRL4^62^. Additionally, the PI3K-AKT-mTOR pathway can be regulated by components of the Hippo pathway, among which YAP downregulates phosphatase and tensin homologue (PTEN), a negative regulator of PI3K-AKT signalling^63^.

OSU-03012, an ATP-competitive inhibitor of PAK, can inhibit VS cell growth and promote apoptosis. Recently, the combination of the PI3K inhibitor pictilisib and the PAK inhibitor PF-3758309 was found to promote cell cycle arrest in mouse and human merlin-deficient Schwann (MD-SCs) cells and to promote apoptosis in mouse MD-SCs^64^. This study is highly instructive for further exploration of targeted therapies for PI3K-AKT-mTOR pathway. Among mesothelioma cell lines, the merlin-negative cell line is sensitive to rapamycin, a specific mTOR inhibitor^65^. However, the efficacy of everolimus (RAD001), a derivative of rapamycin, in *NF2*-associated VS patients has been controversial^38^. In two clinical trials related to neurofibromatosis type 2 VSs (NCT01419639 and NCT01490476), after the use of everolimus, neither the tumor size nor the hearing status significantly improved in children or adults^66,67^. In a single-arm phase II trial involving 59 MPM patients, everolimus also yielded less favourable outcomes than did other agents (NCT00770120)^68^. Similarly, an inhibitor of both PI3K and mTOR, Samotolisib (LY3023414), displayed limited single-agent activity in second-line treated mesothelioma (NCT01655225)^69^.

#### RAS

Several lines of evidence suggest a close relationship between merlin and the rat sarcoma-causing gene (*RAS*), among which merlin can inhibit the RAS-mediated signalling pathway. Moreover, overexpression of merlin can counteract Ras-induced transformations^70^. The anti-Ras function of merlin is believed to involve its N-terminus and C-terminal structural domains, which are required for its tumor suppressor activity^71^. The FERM domain was proven to interact directly with Ras^72^, but whether the antitumour effect of *NF2* is exerted through this interaction has yet to be determined. Recently, it has been shown that *NF2* deletion in thyroid tumors can synergize with *RAS* mutation to increase MAPK signalling^73^. Moreover, p120RasGAP (also known as RasGAP), a well-known negative regulator of Ras, can interact with FERM and the tail domains of merlin, which may be involved in the negative regulation of Ras by *NF2*^72^. Merlin also suppresses the activation of Rac, which is a member of the Rho family of GTPases and can regulate cell movement. Once Rac is inhibited by merlin, Raf and MEK fail to be phosphorylated by PAK, subsequently interfering with Ras-to-MEK signalling^70^. Interestingly, PAK1-3, especially PAK2, can increase the hyperphosphorylated form of merlin, resulting in the inactivation of merlin as well as the inhibition of the Ras signalling pathway^43,74^. Notably, unphosphorylated merlin can inhibit PAK activity, indicating that feedback may exist between PAK and merlin^75,76^.

Intriguingly, RAS is thought to be associated with the Hippo pathway. On the one hand, RAS is influenced by the Hippo pathway, which is a transcriptional target of YAP-TEAD1 and includes three kinds of RASs. Silencing of YAP in Cal62 (KRAS-G12R, *NF2*-null) cells decreased the mRNA levels of all RAS isoforms. Pharmacologic disruption of YAP-TEAD with verteporfin can block *RAS* transcription and signalling and inhibit cell growth^73^. On the other hand, mutant *KRAS* extends to activate the apoptotic MST2-LATS1 serine/threonine-protein kinase 3/STK3-LATS1 pathway by binding to the tumor suppressor *RASSF1A* (Ras association domain-containing protein 1)^77^, which is closely related to the Hippo pathway. Notably, *NF2* and *KRAS* are mutually exclusive, indicating that these genes interact to participate in mesothelioma tumorigenesis^78^. However, further investigations are needed to determine whether these two factors synergize to promote tumor cell proliferation. In terms of its clinical efficacy, when tested on patients with unresectable mesothelioma in a phase II study, the use of sorafenib, a potent inhibitor of the RAS/RAF/MEK pathway, yielded disappointing results. No statistically significant difference in median overall survival was observed between pretreated and chemo-naive patients^79^. To date, divarasib (GDC-6036), sotorasib and the most recently reported Pan-KRAS inhibitor have been explored for the treatment of multiple solid tumors^80–82^, suggesting the feasibility of employing KRAS inhibitors in mesothelioma treatment.

#### FAK

FAK is a cytoplasmic protein kinase that plays a critical role in controlling cell adhesion, invasion, and migration. It has been described in the literature that merlin can inhibit the invasiveness induced by FAK overexpression. Conversely, when Merlin was re-expressed in *NF2*-null mesothelioma cells, the level of FAK markedly decreased^83^. Merlin also attenuates FAK phosphorylation at Tyr397, which functions as a binding site for Src and the p85 subunit of PI3K, resulting in decreased invasiveness^83^. In phase I studies, the FAK inhibitor GSK2256098 was shown to prolong progression-free survival (PFS) in MPM patients with low expression of merlin^84^. Paradoxically, another double-blind randomized phase II study evaluating the FAK inhibitor defactinib as a maintenance agent after first-line chemotherapy showed no significant difference between the two groups^85^. The reasons for these contradictory conclusions are also worth considering.

#### Merlin/*NF2*-Lin28B-Let-7

There are two forms of mammalian Lin28: Lin28A and Lin28B. Both of these proteins have been strongly implicated in several human primary tumors^86^. Lin28B can bind to specific regions of merlin^87^ and inhibit the biosynthesis of let-7 microRNAs (miRNAs) by silencing oncogenes, such as *MYC* and *RAS* (Fig. 3)^88,89^. With low cell density, phosphorylated merlin fails to bind to Lin28B, which reduces the maturation of pri-let-7 miRNAs in the nucleus, and proteins that promote cell growth further accumulate. In contrast, when cell contact is inhibited, Lin28B binds to nonphosphorylated merlin outside the nucleus, after which mlet-7 expression increases. This resulted in the inhibition of cell proliferation. The regulation of the merlin/*NF2*-Lin28-let-7 axis is not affected by YAP1/TAZ and occurs independently of the Hippo pathway^87^.

**Fig. 4:**
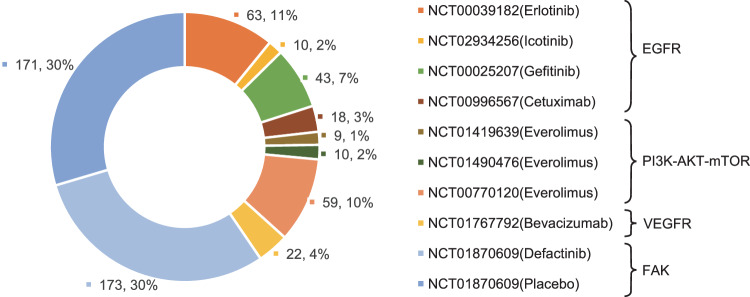
Summary of the clinical trials for *NF2*-driven malignancies. Pie chart depicting the targeted therapy for *NF2*-associated tumors. Most of these completed clinical trials are single-arm studies, except for the NCT1870609 trial. Related to Table 1.

Notably, due to the lack of hotspot mutations, indirectly targeting downstream activation signals driven by *NF2* inactivation might be an alternative strategy. Nevertheless, decades of effort have been expended, and the effectiveness of targeted therapy for *NF2*-associated tumors has been controversial (Table 1, Fig. 4)^35,51,66–68,85,90–94^, highlighting new perspectives on the treatment of *NF2*-deficient tumors.

**Table 1:** Information about clinical trials of *NF2*-related tumors

Molecular Target	Treatment	ID	Disease	EE	Study Type	Primary end point	Conclusion	References
EGFR	Erlotinib		NF2-related progressive VS	11	Retrospective study	Radiographic and hearing response rate	A possible modest treatment response.	^90^
		NCT00039182	MPM	63	A single-arm phase II clinical trial	One-year survival and median overall survival	Single-agent erlotinib was not effective in MPM	^91^
	Icotinib	NCT02934256	NF2-related progressive VS	10	A single-arm phase II clinical trial	Radiographic response	10% radiographic response, 43% hearing response	^92^
	Gefitinib	NCT00025207	Malignant mesothelioma	43	A single-arm phase II clinical trial	The percentage of patients who remain alive and progression-free 3 months	Failed to meet the primary endpoint.	^93^
	Cetuximab	NCT00996567	MPM	18	A single-arm phase II trial	Progression-free survival rate at 18 weeks	The trial was interrupted.	^94^
Ras/MEK/ERK	Selumetinib	NCT03095248	NF2-related VS	34	A phase II trial	Change in hearing response at 24 weeks	Ongoing	
	Brigatinib	NCT04374305	NF2-related progressive tumors	100	A multi-arm phase II trial		Ongoing	
PI3K-AKT-mTOR	Everolimus	NCT01419639	NF2-related progressive VS	9	A phase II study	Objective response rate	No response	^66^
		NCT01490476	NF2-related progressive VS	10	A single-arm phase II trial	Tumor shrinkage	No response	^67^
		NCT00770120	MPM	59	A single-arm phase II trial	Progression-free survival	Limited clinical activity.	^68^
COX	Aspirin		VS	1251(488 + 347 + 86 + 330)	Meta-analysis		No sufficient evidence for aspirin in VS patients.	^51^
		NCT03079999	VS	300	A phase II Trial	Progression-free survival	Ongoing	
VEGFR	Bevacizumab	NCT01767792	NF2-related progressive VS	22	A phase II study	Hearing response	An obvious improvement	^35^
FAK	Defactinib	NCT01870609	MPM	344	a phase II study	Progression-free survival and overall survival	No statistical differences	^85^
Hippo	Pevonedistat	NCT03319537	MPM	9	A phase I & II study	Complete response, partial response or stable disease at 18 weeks	Under evaluation	

*EE* estimated enrollment, *NF2* neurofibromatosis type 2, *VS* vestibular schwannoma, *MPM* malignant pleural mesothelioma.

### Cross-cancer mutation patterns related to *NF2*

The cross-cancer mutation pattern, encompassing cooccurring and mutually exclusive driver gene mutations, can indicate a collaborative relationship or a lethal interaction during tumorigenesis. Cooccurring mutations commonly present in tumors may activate complementary oncogenic pathways, representing distinct biological aspects of cancer. Conversely, artificially induced mutations in mutually exclusive genes tend to induce tumor cell senescence or apoptosis^95^. As mentioned earlier, focusing solely on one mutation and downstream pathways has not proven effective in identifying necessary treatments. These findings suggest that co-occurrence and mutual exclusion occur in tumors and might influence treatment response; further exploration of these processes is warranted in the therapeutic field.

#### *NF2* and *CDKN2A/B*

The *CDKN2A/B* locus is located close to chromosome 9p21. This locus encodes p14^ARF^, p15^INK4b^ and p16^INK4a^ and actively participates in the negative regulation of the cell cycle^96^. According to a finding by Peyre et al., homozygous and heterozygous *Cdkn2a/b* deletions together with biallelic *Nf2* inactivation contribute to increased meningioma frequency with a shorter latency in mice, while single *Cdkn2a/b* inactivation hardly leads to tumor development, indicating that *Nf2* and *Cdkn2a/b* cooperate to promote meningioma progression^96^. In line with these findings, a study revealed that the additional loss of *Cdkn2a/b* in *PGDStv-a*, RCAS-PDGF-B, *and AdCre*; *Nf2*^flox2/flox2^ mice resulted in a greater incidence of Grade II and Grade III meningiomas^97^. Additionally, in comparison with p53 mice, conditional *Nf2*; *Ink4a/Arf* mice exhibit increased tumor invasion and shorter survival^21^. Notably, research on the genetic status of peritoneal mesotheliomas has shown 13% homozygous *CDKN2A* deletions together with hemizygous *NF2* loss. This expression tends to be a negative prognostic factor for both progression-free survival and overall survival, independent of patient age, peritoneal cancer index, completeness of cytoreduction, and extent of invasion^98^. Based on MPM datasets from The Cancer Genome Atlas (TCGA) (*n* = 86) and the Memorial Sloan Kettering-Integrated Mutation Profiling of Actionable Cancer Targets (MSK-IMPACT, targeted screen) (*n* = 61), the percentages of patients with combined alterations in *CDKN2A/B* and *NF2* were 8% (*n* = 7) and 5% (*n* = 3), respectively, which also showed a significant association with poor survival in MPM patients^99^. This means that cooccurring mutations in *NF2* with *CDKN2A/B* may have synergistic effects on tumor incidence and malignancy.

#### *NF2* and *BAP1*

BRCA-associated protein 1 (BAP1) is a member of the deubiquitinase (DUB) family of proteins and acts as a tumor suppressor gene whose mutation occurs in multiple human cancers, especially mesothelioma, uveal melanoma, and clear cell renal cell carcinoma^100^. Inactivation of *BAP1* is often accompanied by disruption of *NF2* and *CDKN2A/B*^101,102^. In a study by Kukuyan et al., MM was rare in purebred mice in which *Nf2* alone was knocked out (2 of 15), while when *Nf2* was combined with *Bap1*, the incidence of MM reached 16.6% (7 of 42)^103^. Furthermore, in compound conditional knockout (CKO) mice with simultaneous inactivation of *Bap1* and *Nf2*, the incidence of HCC (hepatocellular carcinoma) and ICC (intrahepatic cholangiocarcinoma) was greater (28 of 42, 66.7%), while the tumorigenesis of MM occurred earlier than that of other types of tumors in these mice, indicating the potential role of cooccurring mutations in tumorigenesis and the development of cancer types^103^. Approximately 8% (*n* = 7) of the 86 MPM patients and ~5% (*n* = 3) of the 61 MPM patients had combined alterations in *BAP1* and *NF2* according to the TCGA and MSK-IMPACT datasets. Among these cases, this combination may be responsible for the lower hazard ratio, although additional samples are needed for a significant conclusion about this genotype. This combined loss of *BAP1* and *NF2* also results in greater sensitivity to pemetrexed and palbociclib than does the loss of *CDKN2A/B*, which offers an ideal approach for guiding stratified treatment in MPM^99^.

#### *NF2, BAP1* and *CDKN2A*

Kukuyan et al. induced specific loss of *Bap1*, *Nf2*, and *Cdkn2a* and the combination of two or more proteins in mesothelial cells by injecting Adeno-Cre into homozygous single-gene CKO mice and homozygous compound CKO mice^103^. The incidence of MM was 84.6% (22 of 26) in *Bap1*; *Nf2*; *Cdkn2a* (triple)-CKO mice, approximately six times greater than that in mice in which *Nf2* was knocked out alone. Furthermore, differences in the stem cell characteristics of mesothelial cells were more evident in triple-CKO mice than in other mice. In a way, tumorigenesis requires accumulated genetic and epigenetic alterations^103^. Similar findings were also reported by Badhai et al., in which the combined loss of *Bap1*, *Nf2*, and *Cdkn2a/b* (BNC) led to mesothelioma in all mice of the cohort^102^. Additionally, they reported that the BNC mesothelioma model is very similar to the immunoinflammatory phenotype induced by asbestos. The immune cell composition in BNC closely resembles that in human mesothelioma with *BAP1*, *NF2*, and *CDKN2A* loss—M2 macrophages, T cells, and B cells make up a significant proportion of the leukocyte population^102^. Moreover, genetic alterations in *BAP1*, *NF2* and *CDKN2A/B* in the TCGA and MSK-IMPACT cohorts related to MPM were approximately 13% and ~3%, respectively, and these alterations were significantly associated with poor survival in the TCGA cohort^99^. Overall, cooccurring deficiencies in *BAP1*, *NF2*, and *CDKN2A/B* might play an instructive role in tumor immunity and are closely related to patient prognosis.

#### *NF2* and *LATS2*

Regarding cooccurring *NF2* and large tumor suppressor 2 (*LATS2*) mutations, Tranchant et al. defined a new molecular subgroup of MPM, named the C2^LN^. C2^LN^ is characterized by a cooccurring mutation in the *LATS2* and *NF2* genes and is associated with a poor prognosis. Interestingly, this subgroup appears to be specifically associated with MPM, with low mutation frequencies in other human malignancies^104^. However, when the C2^LN^ MPM subgroup exhibited decreased phosphorylation of YAP, it displayed a phenotype resistant to verteporfin (a potent YAP inhibitor). Notably, the C2^LN^ MPM subgroup showed improved drug sensitivity to mTOR inhibitors, which might be due to the hypophosphorylation of mTOR^104^. Interestingly, these findings contrast with the observed upregulation of mTOR expression in Merlin-deficient or *LATS1/2-*deficient tumors^60,105^. Hence, questions remain about the changes in molecular events in *NF2-* and *LATS2*-mutant MPMs, as well as the use of mTOR inhibitors in this specific subtype.

#### *NF2* and *PTPRJ*

DEP-1 (density-enhanced phosphatase-1, encoded by *PTPRJ*) is a tumor suppressor that plays a role in meningioma. The depletion of both protein-tyrosine phosphatase receptor type J (*PTPRJ)* and *NF2* led to an altered cell shape in vitro, suggesting reduced adherence and spreading. Neither the cooccurring deficiency of merlin nor DEP-1 had a combined functional effect on cell proliferation or viability in vitro. However, in vivo studies revealed that the combined depletion of both molecules promoted meningioma tumorigenesis compared with the single loss of *Nf2* in *Nf2*-floxed mice^106^. It is possible that other molecular alterations may occur later to functionally cooperate with the combined loss of DEP-1 and merlin and contribute to the tumorigenesis of meningioma in vivo. However, the cellular mechanisms underlying the combined effects of DEP-1 and merlin loss, together with their biological impact, remain to be identified.

#### *NF2* and *KRAS*

The first exploration of the relationship between *NF2* and *Ras* could date back to 1994, when Tikoo et al. reported that the overexpression of full-length *NF2* could reverse the *Ras*-induced malignant phenotype^71^. Moreover, in an examination of the transcriptomes from the TCGA-MPM cohort, *KRAS* mutations also exhibited mutually exclusive effects on *NF2* mutations^78^, indicating that they may have a similar impact as *NF2* deficiency in MPM. Notably, the role of oncogenic *KRAS* mutations in the TME has gradually been revealed^107,108^. By influencing several TME components, such as neutrophil chemokines, granulocyte macrophage colony-stimulating factor, vascular endothelial growth factor, and many cytokines, including IL-8, IL-10, IL-17, and TGFβ1, *KRAS* mutations can regulate the recruitment, activation, and differentiation of immune cells^108^. Therefore, it is worth considering whether *NF2* mutation can regulate the TME through its effect on *KRAS*. To date, encouraging results have been obtained in clinical trials of drugs directly targeting KRAS, especially KRAS-G12C inhibitors^109^. Considering these findings, the use of KRAS-G12C inhibitors, such as AMG510 (sotorasib) and MRTX849 (adagrasib), could be effective against *NF2*-related tumors and warrants future exploration.

#### *NF2* and *TRAF7*

Secretory meningiomas, which make up approximately 3% of all meningiomas, are characterized by combined Kru ¨ppel-like Factor 4 (*KLF4)*^K409Q^ and *TRAF7* mutations^110^. Interestingly, this meningioma subtype was significantly correlated with a lack of *NF2* mutations, which suggests that novel mutations in *KLF4* and *TRAF7* may both be mutually exclusive to alterations in *NF2*^111^. This finding is also consistent with the finding that *TRAF7* is deficient in non-*NF2*-mutated intraventricular meningiomas (IVMs)^112^. Moreover, in MPM and meningioma, *TRAF7* and *NF2* also exhibit mutually exclusive relationships, which suggests that they are involved in a common signalling cascade^9,113^. However, the downstream signalling pathways involved have not been fully elucidated.

**Fig. 5:**
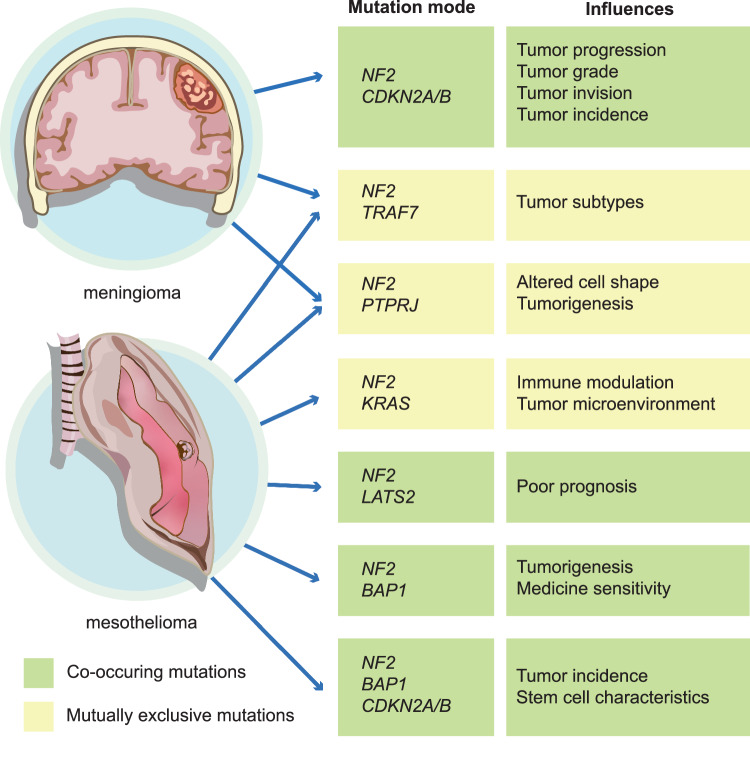
Cross-cancer mutation patterns related to *NF2.* To date, cross-cancer mutation patterns related to *NF2* are strongly associated with mesothelioma and meningioma. The green background represents cooccurring mutations, and the yellow background represents mutually exclusive mutations.

Taken together, the above evidence highlights that cross-cancer mutations in *NF2*-related patients are associated with unique tumor characteristics and should be considered before clinical treatment decisions are made (Fig. 5).

## Metabolic roles of *NF2* in tumors

Over time, the metabolic reprogramming of tumors has been continuously investigated^114,115^. Compared with previous statements that the restriction of energy production in tumors tends to be the key driver for metabolic programming^116^, the availability of reduced nicotinamide adenine dinucleotide (NAD+) seems to be more limiting than energy for the proliferation of tumor cells^117,118^, indicating that the pathways by which metabolites influence tumor cells are also constantly being enriched. Moreover, studies on the metabolic reprogramming of *NF2*-deficient malignancies have advanced. Compared with those of wild-type *NF2*, both *Nf2*-null mouse embryo fibroblasts (MEFs) and mouse Schwann cells exhibit generalized metabolic alterations, with merlin mutants (NF2m) also playing a role in cellular metabolism^119,120^. Changes in steady-state metabolite levels, including elevated tricarboxylic acid (TCA) cycle metabolites, increased levels of nicotinate metabolites (NAD+, NADH, and NADP) and pantothenate metabolites, decreased levels of glycolysis and amino acid metabolites, and upregulated glutaminolysis, have been observed in *NF2-*deficient tumors^119^. However, the underlying mechanisms by which *NF2* deletion leads to metabolic reprogramming remain unclear.

### Carbohydrate metabolism

In terms of glucose metabolism, decreased levels of metabolites associated with glycolysis can be observed in *NF2*-deficient Schwann cells^119^. Intriguingly, the deletion of *NF2* induces the activation of the mTOR and RAS pathways, which are related to hypoxia-inducible factor 1α (HIF-1α), a factor that can enhance the expression of several enzymes in the glycolysis pathway^121,122^. Moreover, according to speculation, the phosphorylation of YAP in the Hippo pathway is inhibited in merlin-null cells, which subsequently contributes to the activation of downstream target genes; among these findings, the YAP-TEAD complex can promote glucose uptake and glucose metabolism by affecting glucose transporter 3 (GLUT3)^123^. These findings are also consistent with the finding that YAP/TAZ engage the PI3K-AKT pathway to promote glycolysis in *NF2-*mutant kidney tumors^124^. Paradoxically, the above studies contradict the observation that the absence of *NF2* leads to a decreased level of glycolytic metabolism, indicating that a cell context-specific metabolic rewiring system might exist and awaits exploration.

### Lipid metabolism

Emerging evidence has suggested a potential link between *NF2* deficiency and lipid metabolism, which tends to be the prominent metabolic feature in *NF2*-deficient cells^119^. Compared with wild-type cells, *NF2*-deficient fibroblasts and Schwann cells exhibit significant elevations in fatty acid levels associated with lipid metabolism, where the expression of lipogenesis-related genes is significantly elevated^119^. In other experiments, a decrease in the expression of genes such as fatty acid synthase (FASN) and acetyl-CoA carboxylase 1 (ACC1, encoded by *ACACA*) was observed when the cell density increased, while the expression of these genes significantly increased with *NF2* knockout, which further accounts for the association between *NF2* and lipid metabolism^125^. These cells characterized by loss of *NF2* are sensitive to small interfering RNA (siRNA) or small-molecule inhibitors of FASN, which not only demonstrate cellular alterations in lipid metabolism but also suggest that drugs such as FASN inhibitors may have certain clinical effects on *NF2*-deficient cells^119^. In colon, breast, and prostate cancer and human MM cells, FASN inhibitors have been shown to inhibit cell proliferation^126,127^. It is possible that this effect on lipid metabolism is caused by the activation of mTOR, which in turn upregulates sterol regulatory element binding protein 1 (SREBP1) and lipin1^119^.

It is intriguing that statins, small molecule inhibitors of 3-hydroxy-3-methyl-glutaryl-CoA (HMG-CoA) reductase and often applied for reducing cholesterol levels, have been reported to inhibit mesothelioma growth both in vitro and in mouse xenografts^128–130^. Lovastatin reduced cell migration and cell viability in an *NF2*-mutant mesothelioma cell line (ACC-MESO-1), possibly through the induction of mTOR-independent autophagy^128^. Another study on twelve mesothelioma cell lines indicated that cells harboring *NF2* and/or *LATS2* mutations were more sensitive to statins than those harboring *BAP1* mutations^129^. The exploration of the mechanism by which statins impact mesothelioma cells is ongoing. Alterations in the levels of mevalonate (the product catalyzed by HMG-CoA) and farnesyl (a critical mevalonate metabolite) were identified in lovastatin-related research, while cholesterol was less likely to be a factor^130^. It is plausible that statins could affect downstream targets, including the Rac/phospholipase C/inositol 1,4,5-triphosphate axis, and the acylation of guanosine triphosphate-binding proteins^128,130^. Moreover, statins, such as fluvastatin and simvastatin, have also been demonstrated to indirectly inhibit the activation of YAP-TEAD in *NF2*-deficient mesothelioma cells^129^.

Moreover, cellular lipid metabolism is closely linked to ferroptosis. Unrestricted lipid peroxidation, which leads to subsequent plasma membrane rupture, is a major contributor to ferroptosis^131^. The relationship between tumor suppressors and ferroptosis has currently been discussed, and *p53* and *BAP1* have been shown to be responsible for ferroptosis resistance^132,133^. However, in contrast to previous findings, Wu et al. reported that inactivation of *NF2* could promote ferroptosis sensitivity. Mechanistically, *NF2* inactivation leads to a decrease in LATS1/2 expression, and increased YAP/TAZ activity is detected in these cells. YAP/TAZ further regulates downstream genes such as transferrin receptor 1 (TFRC) and acyl-CoA synthetase long chain family member 4 (ACSL4), both of which are crucial ferroptosis modulators. Therefore, mutations in *NF2* could be good predictors of responsiveness to the induction of ferroptosis^61^.

### Amino acid metabolism

Regarding protein metabolism, a decrease in amino acids, especially glutamine metabolites, was observed in *Nf2*-deficient MEFs and Schwann cells, indicating upregulated glutaminolysis, which could further promote a more active TCA cycle to some extent^119^. Moreover, an increase in mTORC1 expression occurs in merlin-inactivated cells^59^ and is involved in the regulation of protein synthesis and amino acid synthesis and transportation. In terms of amino acids, mTORC1 is mainly associated with the upregulation of asparagine biosynthesis in colorectal cancer cells, but whether it can directly regulate glutamine metabolites has not been determined^121^. Intriguingly, glutamine metabolism is highly important for mTORC1 activation^134^. On the one hand, glutamine together with leucine enhances glutaminolysis, contributing to the activation of mTORC1. On the other hand, glutaminolysis activates mTORC1 by stimulating GTP loading of RagB (a part of Rag GTPases)^135^. However, further investigations of the impact of *NF2* on amino acid metabolism are needed to determine whether this effect occurs through the mTOR pathway. In terms of the *RAS/KRAS* pathway, *KRAS*-mutant tumors exhibit glutamine-related metabolic reprogramming, which elevates the expression of enzymes involved in glutaminolysis^136^. Notably, *NF2* and *KRAS* are mutually exclusive, indicating that they might share similar downstream events, including glutamine metabolism. Interestingly, macropinocytosis is dependent on oncogenic *RAS* expression, and further study indicated that *KRAS*-transformed cells are capable of utilizing macropinocytosis to supply intracellular amino acids, including glutamine^137^, while *NF2*-deficient cells are unable to utilize macropinocytosis to obtain exogenous glutamine, although with high macropinocytotic activity^138^. Moreover, under acute nutrient deprivation, autophagy in *KRAS*-driven cancer cells can divert nutrients, including glutamine and glutamate, to meet metabolic demands^139^, but conversely, loss of merlin leads to attenuated autophagy^138,140^. The above evidence suggested that *KRAS*-driven cells can acquire glutamine through multiple ways, none of which are fully underutilized in *NF2*-related cells, further indicating that *NF2*-mutant cells tend to be glutamine deficiency. To date, targeting glutamine metabolism enzymes, such as glutaminase (GLS1, an enzyme that restricts the conversion of glutamine to glutamate and its cataplerotic entry into the TCA cycle), in combination with chemotherapy is promising for suppressing tumor growth^141,142^. It is possible that after subsequent exploration, targeting glutamine metabolism could become a new direction for the treatment of *NF2*-related tumors.

### Nicotinate metabolism

Increased metabolism of nicotinate, such as NAD+ and NADH, which are involved in mitochondrial electron transport (ETC) trains, was observed in *NF2*-deficient tumors, indicating that there might be an enhanced capacity for energy production as well as other NAD+-related metabolic activities, including glycolysis, glutaminolysis and fatty acid oxidation^119,143^. Moreover, Merlin-mutant mouse Schwann cells exhibit increased expression levels of SIRT2 (sirtuin 2, an NAD+-dependent protein deacetylase), which may also reflect upregulated nicotinate metabolism^144^. It has been universally established that NAD(H) and NADP(H) serve as carriers that participate in reduction and oxidation reactions and are widely involved in various cellular metabolic alterations^145^. The NAD+/NADH ratio also plays an essential role in aspartate synthesis, which is a limiting factor for tumor growth^117,146^. Moreover, Luengo et al. suggested that cells engage in aerobic glycolysis when the need for NAD+ exceeds the demand for ATP, and the availability of NAD+, rather than ATP, affects cell proliferation^118^. Overall, it is possible that nicotinate metabolism may centrally underlie the alteration of glycolysis and amino acid metabolism or may act as a mirror of cellular energy production in *NF2*-mutant cells.

### Nucleotide metabolism

It is widely acknowledged that nucleotide metabolism, as a foundation of nucleic acid constitution, has important implications for uncontrolled proliferation, chemotherapy resistance, immune evasion and metastasis in cancer cells^147^. Consistent with the above observations of a close link between *NF2* and glutamine catabolism, a pivotal carbon source for nucleotide metabolism, cancer cells with reduced *NF2* expression are highly sensitive to drugs such as cytarabine, oxaliplatin, and 5-fluorouracil, which inhibit DNA synthesis^148^. The mTOR complex, especially mTORC1, can regulate pyrimidine and purine synthesis via the control of the trifunctional multidomain enzymes CAD (carbamoyl-phosphate synthetase 2, aspartate transcarbamylase, and dihydroorotase) and ATF4 (activating transcription factor 4)^149^. Hippo-YAP can also reprogram glutamine metabolism by regulating glutamine synthetase, subsequently influencing nucleotide synthesis^123^. Our ongoing research revealed that *NF2* deletion may mediate the de novo synthesis of pyrimidine nucleotides via the Hippo–YAP axis, suggesting that small molecule drugs targeting de novo pyrimidine synthesis may be an effective approach for treating *NF2* mutant tumors^150^. However, a great deal of research is still needed to understand how *NF2* affects nucleotide metabolism and what potential therapeutic targets are involved.

## *NF2* and immunotherapy

Immunotherapy has revolutionized cancer treatment due to its amazing clinical efficacy. *NF2* deficiency leads to a tumor milieu characterized by immunosuppression, which is orchestrated by multiple mechanisms. For instance, changes in metabolite composition can inhibit the infiltration and function of immune effector cells, such as T cells and natural killer cells, while promoting the accumulation of immunosuppressive cells, such as regulatory T cells (Tregs) and myeloid-derived suppressor cells (MDSCs). *NF2* may also indirectly influence immune checkpoints, playing a subtle yet significant role in the evasion of immune surveillance. In recent years, several studies have shown that *NF2* deficiency is inextricably linked to the tumor immune microenvironment (TIME)^30,47,151^. Therefore, exploring the application of immunotherapy in *NF2*-related tumors is promising.

### Innate immunity

After investigating the distribution of immune subtype models across the TCGA MPM cohort, Yang et al. reported that high protein levels of *NF2* were more closely related to the IFN-gamma dominant, inflammatory, and TGF-beta immune subtypes than low merlin levels were. The lymphocyte-depleted and wound healing subtypes are correlated with low protein levels of NF2^47^. It seems that MPM in the presence of merlin contains more abundant inflammatory factors. Moreover, regarding immune cells involved in innate immunity, the enrichment of macrophages in MG1 tumors was confirmed^30^. However, research on other characteristics of innate immunity, including natural killer (NK) cells and monocytes, in *NF2*-related tumors is still lacking, and additional explorations of *NF2*-associated tumors and intrinsic immunity are still scarce.

### Adaptive immunity

#### B lymphocytes

A potential loss of antibody-mediated humoral immunity has been discovered in *NF2*-null MPM, and subsequent studies have shown that *NF2* expression is positively correlated with the gene expression of CD20 (a B lymphocyte-specific membrane protein). Moreover, MPM patients with low *NF2* expression are characterized by high plasma B-cell infiltration and improved overall survival^47^. Intriguingly, elevated B-cell infiltrates have been shown to play a role in predicting the response to immune checkpoint blockade (ICB) therapy and the prognosis of patients^152^. Low *NF2* levels and high plasma B-cell infiltration may serve as predictors of the response to ICIs in MPM patients in the future. A team recently identified a key immune checkpoint in B lymphocytes, TIM-1. Targeted inhibition of TIM-1 in B cells enhances the antitumour responses of CD8^+^ and CD4^+^ T cells and inhibits tumour growth^153^. However, the role of B lymphocytes in *NF2*-associated tumors has not been determined. Additionally, whether immune checkpoints targeting B cells function in MPM requires further investigation. Intriguingly, in contrast to the positive correlation between *NF2* and CD20 expression in MPM described above, a limited percentage (11%) of CD20^+^ tumor-infiltrating lymphocytes (TILs) were observed in meningiomas and schwannomas compared with other T lymphocytes, such as CD68^+^, CD3^+^, or CD8^+^ TILs^151^. However, whether these alterations occur through the selective activation of different downstream signalling pathways has not been determined, which highlights the need for further studies to understand the complex biology underlying this phenomenon.

#### T lymphocytes

*NF2* mutation in MPM was also associated with T lymphocyte infiltration. NF2m tended to reverse the enrichment of CD4^+^ and CD8^+^ T lymphocytes in a STING-initiated murine model^120^. In addition, in a study by Yang et al., tumor-infiltrating CD8^+^ T cells were found to be more enriched in MPM harboring LATS1/2 mutations than in *NF2*-mutant MPM^47^. Further analysis of lymphocytes from *NF2*-related patients (including 10 meningiomas and 10 schwannomas) revealed that there was a sparse to moderate presence of CD68^+^, CD3^+^, or CD8^+^ TILs at low microscopic magnification (100×)^151^. However, interestingly, in MPM, sarcomatoid/biphasic samples, which are closely related to *NF2* deficiency, were characterized by increased CD8 + T lymphocytes^154^. Furthermore, as a vital immune checkpoint protein, programmed death ligand 1 (PD-L1), binds to programmed death 1 (PD-1) on T cells, thereby contributing to cancer immunosuppression^155^. PD-L1 expression was greater in sarcomatoid/biphasic MPM than in epithelioid MPM^154^. Nevertheless, among 50 MPM patients, a significant correlation at the protein level was not detected between *NF2* mutation status and PD-L1 expression^47^. It was prudent to infer that the high PD-L1 expression may be attributed to other molecules, but additional evidence is needed.

In addition to the abovementioned reports on T cells, B cells and immune checkpoints, few reports have explored other adaptive immune cells, including Tregs and MDSCs, which deserve further study.

### Potential mechanisms

**Fig. 6:**
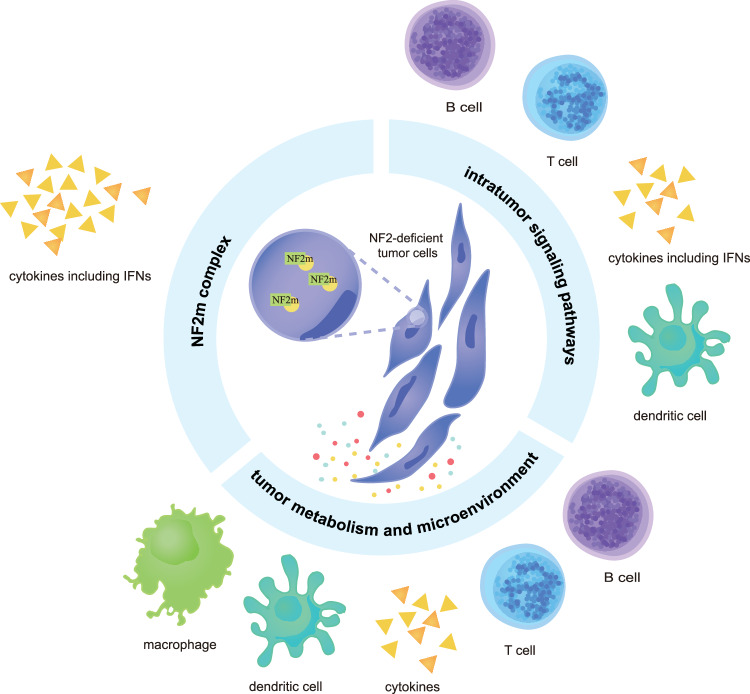
Interaction between *NF2* and tumor immunity. We propose three main approaches for determining the mechanism by which the loss of *NF2* affects the regulation of immune cells (such as T cells, B cells, dendritic cells, and other cytokines, including IFNs): (1) through intratumor signalling pathways; (2) through the NF2m complex; and (3) through the tumor microenvironment.

We propose three main approaches for determining the mechanism by which the loss of *NF2* affects the regulation of immune cells (Fig. 6): (1) *NF2* impacts tumor immunity by regulating the expression of specific molecules and signalling pathways in tumors, and (2) Merlin mutants (NF2m, with missense mutations in the N-terminal FERM domain) can directly influence tumor immunity. (3) *NF2* alters immune cell infiltration by changing the TME.

#### *NF2* affects immune cells through intratumor signalling pathways

##### Hippo pathway

The Hippo pathway is closely related to immunoregulation, especially through MST1 and YAP/TAZ. The kinase MST1 functions as an important regulator of T-cell adhesion, migration, proliferation, and apoptosis, as well as in dendritic cells. In vitro, the Nore1B/Mst1 complex inhibits the proliferation of naive T cells. *Mst1*-null mice exhibit fewer naive peripheral T cells, while the effector/memory T-cell cohort is similar to the wild-type cohort, which also indicates that MST1 participates in maintaining circulating naive T lymphocytes^156^. When *Mst1* and *Mst2* were double knocked out in mice, the migratory ability of single-positive thymocytes together with the ability of T cells were strongly inhibited, suggesting that MST participates in the migration of T cells^157^. Moreover, the Hippo pathway may impact cancer cells through the alteration of cytokines, including type I interferons (IFNs). IFNs are polypeptides that participate in segregating viral infections and modulating immune responses. Melin can act on downstream molecules through direct or indirect regulation of LAST1/2 and YAP/TAZ to further influence the production of IFNs. For example, in the Hippo pathway, LATS1/2 can stimulate the host TLR-MYD88/TRIF nucleic acid-sensing pathway, thereby inducing the production of type I IFN^158^. However, the role of the Hippo pathway in the immune system in *NF2*-deficient tumors has not been extensively characterized and deserves further study.

##### cGAS-STING signalling

The cGAS-STING pathway is closely related to antitumour immunity. In the cGAS-STING pathway, cytosolic DNA sensors (e.g., cGAS) bind to aberrant self-DNA or microbial DNA in the cytoplasm to synthesize cGAMP, which further induces the formation of STING. cGAS-STING is a naturally occurring immune protein that mediates the activation of TBK1 and IkB kinase-related kinase ε (IKKε), thereby phosphorylating IRF3. Activated IRF3 coordinates with simultaneously activated nuclear factor κB (NF-kB), further promoting the production of type I interferons (IFNs), proinflammatory cytokines, and chemokines^120^. According to Meng et al., *NF2* deficiency results in compromised activation of TBK1 and IRF3, reflecting the fact that *NF2* deficiency causes a reduced level of cytosolic sensing of RNA analogues and DNA analogues. Additionally, the C-terminal tail of the *NF2* mutant strongly interacted with TBK1 and exerted an inhibitory effect on it.

We speculate that this effect may be a mode of action through which tumor cells suppress immunity. For instance, in the cGAS-STING pathway, merlin can promote nucleic acid sensing by relieving YAP/TAZ-mediated TBK1 inhibition, which has an impact on downstream IFNs^120^ and subsequently influences innate and adaptive immune responses^159^, such as the initiation of Baftf3 DCs and T cells^160^. Furthermore, this pathway is also associated with immune checkpoint inhibitors^161^. During local irradiation (IR), an increase in IFN-L1 production contributes to the upregulation of PD-L1, which suggests that cGAS-STING potentially modulates PD-L1 expression by regulating IFN-L1^162^. Notably, IFNs exert potent immune effects by controlling these tumors, but the clinical efficacy of related therapies still needs to be explored.

##### Other pathways

Pathways, including the PI3K-AKT-mTOR and RAS/KRAS pathways, play crucial roles in shaping the immune microenvironment. PI3K signalling has been shown to participate in the activation of immune cell differentiation and development; the expression of immunoglobulins, chemokines and cytokine receptors; the regulation of phagocytosis; and cell migration^163–165^. This pathway also plays a role in the regulation of PD-L1^163^. Similarly, tumor cells defective in the RAS/KRAS pathway interact with immune cells in the TME by secreting a series of cytokines, such as TGF-β, IL-8, and IL-6, which are involved in macrophage reprogramming and the regulation of Treg differentiation^166^. However, the participation of these pathways in *NF2*-associated tumors has not been extensively characterized.

#### Effects of the NF2m complex on immune cells

In addition to alterations in downstream signalling pathways caused by *NF2* deletion, mutations in *NF2* play a role in the immune system. The cGAS-STING pathway initiates the production of IRF3 (a kind of transcription factor) when cytosolic DNA sensors recognize microbial and aberrant self-DNAs. Subsequently, activated IRF3 induces the formation of an NF2m-containing aggregate in the cytoplasm that harbors abundant endogenous TBK1 (Tank-binding kinase 1), IRF3, and MST1. These NF2m complexes inactivate TBK-1, which negatively feeds back to inhibit the activation of TBK1 and reduce the generation of IFNs [109]. Moreover, these compounds play a role in preventing CD4+ and CD8 + T lymphocyte infiltration induced by SAVI-SRING in melanoma tumors. Increased melanoma growth with decreased T lymphocyte and macrophage infiltration was observed in a mouse model that contains NF2m complexes, suggesting that NF2m induces antitumour immunity^120^.

#### *NF2*-related tumor metabolism affects immune cells in the TME

Growing evidence has established that tumor suppressor genes, including *p53*, *PTEN*, *RB1* and *CDKN2A*, can modulate immune functions, such as regulating Toll-like receptor function, producing cytokines, and regulating immune cell differentiation, synapsis and evasion^167^. Metabolic reprogramming, a characteristic of tumors, is also closely related to tumor suppressor genes. Intriguingly, they tend to regulate metabolism by adjusting downstream signalling pathways rather than modifying enzymes^168^. For example, p53 exerts a negative effect on lipid metabolism through the inhibition of SREBP-1. LKB1 (also known as STK11) influences the expression of downstream molecules by regulating the expression of AMPK and its family kinases, which in turn affects subsequent metabolic reprogramming^169^. Tumor cells affect immunity through metabolic pathways, such as by competing for nutrients or releasing metabolites. Thus, tumor suppressor genes, cancer metabolism and tumor immunity are inextricably interrelated.

Comprehensive metabolic analysis revealed that elevated lipid metabolism may be the key metabolic feature of *NF2*-driven tumorigenesis. Hyperactive lipids in *NF2*-deficient tumor cells compete with immune cells for fatty acid resources, which has important implications for the cell membrane construction of immune cells and other key lipid cell structures^170^. Additionally, the accumulation of lipid metabolites such as long-chain fatty acids, short-chain fatty acids and cholesterol in tumor-infiltrating myeloid cells is associated with immunosuppressive and anti-inflammatory phenotypes^170^. This hyperactive lipid metabolism may result in robust improvements in intracellular lipid peroxidation, further impacting ferroptosis. Notably, *NF2* inactivation increases the sensitivity of cancer cells to ferroptosis^61^, which is inextricably linked to tumor immunity^131^. It is likely that *NF2* deficiency can affect immune cell function through ferroptosis. Ferroptotic cancer cells release high mobility group box (HMBG), mutant KRAS oncoprotein or other damage-associated molecular patterns (DAMPs), leading to increased inflammatory responses in macrophages^171^ and the polarization of macrophages to the M2 phenotype, thereby supporting tumor growth^172^. In tumor cells, anti-PD-L1 therapy promotes lipid peroxidation-dependent ferroptosis. Moreover, anti-PD-L1 agents can synergize with ferroptosis activators (such as erastin and RSL3) to affect tumor growth^173^. It is plausible that the use of immunotherapy combined with ferroptosis activators in *NF2*-deficient tumors has potential in terms of clinical application.

With respect to glutamine metabolism, *NF2* deficiency leads to upregulated glutaminolysis. This altered metabolism results in a decrease in glutamine in the surrounding environment, which is important for cell fate determination and immune responses, such as T-cell proliferation, cytokine production and the transformation of CD4 + T cells to inflammatory cells. By stimulating IL-4, glutamine also functions in mediating M2 macrophage polarization^174^. A lack of glutamine influences immune cells through their differentiation and function in the TME. In addition, another study revealed that glutamine deficiency in conjunction with inhibition of the mTOR1 signalling pathway, both of which occur in *NF2*-deficient cells, increased the release of Rab11-positive exosomes. The release of exosomes plays a role in cell proliferation and turnover, as well as in blood vessel networks^175^. With few side effects, inhibitors of glutamine transporters or glutaminases seem to be effective in vivo^176^. Furthermore, intratumoral glutamine supplementation enhances cDC1-mediated CD8 + T cell immunity, thereby inhibiting tumor growth and overcoming resistance to immune checkpoint blockade^177^. Glutamine supplementation and specific inhibition of glutamine transporters may inhibit *NF2*-associated tumor cells both metabolically and immunologically, which deserves subsequent investigation.

Nucleotide metabolism, a foundation of cell survival and function, has also been demonstrated to be involved in many processes of antitumour immunity, such as immune evasion, tumour growth and metastasis^178^. Both cancer cells and immune cells are predisposed to prefer de novo nucleotide synthesis to the salvage pathway^179^. A growing body of evidence suggests that targeting nucleotide metabolism, including pyrimidine synthesis, can enhance the antitumour response to immunotherapy^180^. Paradoxically, research has shown that altered nucleotide handling might also facilitate tumor immune escape by triggering nucleotide deprivation in immune effector cells^147^. The metabolic crosstalk between cancer cells and immune cells and how this crosstalk impacts immune surveillance and antitumour immunity in MPM warrants further investigation.

### Connections and advances between *NF2-*mutant tumors and immunotherapy

On the one hand, an increased density of scattered lymphocytes was closely associated with *NF2* mutation. On the other hand, patients with scattered lymphocytes exhibited a greater tumor mutational burden (TMB) than patients with other lymphocytes, suggesting that the former had no lymphocyte infiltration. A mutational burden confers susceptibility to immunotherapies to some degree^181^, indicating that *NF2* may function as a predictor of immunotherapy efficacy in meningiomas. The present study revealed a link between mutated genes and the histological subtypes of MPM. *BAP1* deletion is closely related to the epithelioid histotype, whereas *NF2* deficiency is more frequent in the biphasic and sarcomatoid histotypes^25^. Another study revealed that patients in the nonepithelioid group tended to have greater lymphocyte infiltration, and immune checkpoint molecules were more highly expressed in the nonepithelioid group than in the epithelioid group^182,183^. These findings suggested that immune checkpoint inhibitors may have better therapeutic effects on nonepithelioid MPM. As suspected, a phase III clinical trial showed that the concomitant use of ipilimumab (a CTLA-4 inhibitor) and nivolumab (a PD-1 inhibitor) in comparison to the conventional regimen of pemetrexed and platinum, particularly in the nonepithelial subtype of MPM, resulted in a statistically significant improvement in overall survival (OS) (18.1 versus 14.1 months, *p* = 0.002)^184^. These findings shed new light on how subsequent therapeutic dosing of *NF2* might be a biomarker for stratified immunotherapy for mesothelioma.

## Concluding remarks and future directions

As a tumor suppressor gene, *NF2* plays a distinctive role in associated tumors, influencing disease progression, therapeutic approaches, and patient prognosis. Despite the dysregulation of signalling pathways observed in tumors with altered *NF2* expression, targeted therapy is still lacking. Furthermore, concurrent mutations in *NF2* and other genes should be taken into consideration because they invite inquiry into the biological features of these patients and potential therapeutic strategies. To date, the interplay between tumor metabolism and the immune system has attracted increased research attention. Growing evidence also highlights the link between *NF2* and cancer metabolism reprogramming, as well as tumor immunity. However, additional investigations into the underlying mechanisms are crucial for determining whether targeted therapy or immunotherapy could improve prognosis in patients with *NF2*-related tumors.

## Data Availability

The data referenced in this review can be accessed through the following resources numbered in the References section.
